# Short-Term Left Ventricular Remodeling After Revascularization in Subacute Total and Subtotal Occlusion With the Infarct-Related Left Anterior Descending Artery

**DOI:** 10.4021/cr83w

**Published:** 2011-09-20

**Authors:** Ahmet Celik, Nihat Kalay, Hasan Korkmaz, Orhan Dogdu, Omer Sahin, Deniz Elcik, Seyhan Karacavus, Ali Dogan, Tugrul Inanc, Ibrahim Ozdogru, Abdurrahman Oguzhan, Ramazan Topsakal

**Affiliations:** a Department of Cardiology, Elazıg Education and Research Hospital, Elazig, Turkey; b Department of Cardiology, Erciyes University Medical Faculty, Kayseri, Turkey; c Department of Nuclear Medicine, Erciyes University Medical Faculty, Kayseri, Turkey

**Keywords:** Left ventricular remodeling, Total and subtotal occlusion, Left anterior descending artery

## Abstract

**Background:**

Large randomized studies revealed that percutaneous coronary intervention has no clinical benefit in patients with total occlusion. The purpose of this study is to evaluate left ventricular remodelling after PCI for total and subtotal infarct-related left anterior desending artery in stable patients who have not received trombolytic theraphy.

**Methods:**

Sixty stable patients with subacute anterior myocardial infarction who have total or subtotal occlusion in the infarct-related left anterior descending artery were enrolled the study (20 patient in the total-medical group, 20 patient in the total-PCI group and 20 patient in the subtotal-PCI group). All patients’ left ventricular diameters, volumes and ejection fractions measured at admission and after a month.

**Results:**

The necrotic segment number in scintigraphy were similar in three groups. In the total-PCI group, there were significant increases in left ventricular diastolic diameter, left ventricular end-diastolic volume and left ventricular end-systolic volume at first month. A borderline significant increase was observed in LVEDV in the total-medical group at first month. No significant difference was seen in all echocardiographic parameters in the subtotal-PCI group at a month after discharge. The percentage of increase in LVEDV was significantly higher and the percentage of increase in LVESV was borderline significantly higher in the total-PCI group than the other groups.

**Conclusions:**

In stable patients, PCI for total occlusion in the subacute phase of anterior MI causes an increase in LV remodeling. Nevertheless PCI for subtotal occlusion in the subacute phase of anterior MI may prevent LV remodeling.

## Introduction

The fibrinolytic therapy and primary percutaneous coronary intervention (PCI) is early reperfusion strategies to treat ST segment elevation myocardial infarction (MI). However these strategies cannot be performed in about one third of patients because of late presentation [1]. Therefore, management of patients experiencing late phase MI is an important clinical issue. Previous studies showed that PCI had no clinical benefit for patients with total occlusion of the infarct-related coronary artery [2-4]. However, based on the open artery hypothesis, some studies suggested that PCI applied to total occlusion may improve LV function [5-9].

Because of great heterogeneity in available studies, clinical decisions are still obscure and controversial in a specific patient group with total occlusion. Current guidelines do not suggest PCI for total occlusions in an infarct-related coronary artery in stable patients. However, most interventional cardiologists are willing to perform PCI for total occlusion. Therefore, further data are needed concerning efficacy of PCI to total occlusion in a specific patient group. It was not clarified that PCI for subtotal infarct-related artery has a favorable effect on LV remodelling and functions.

In this study, we aimed to demonstrate the effect of PCI or medical therapy on short-term left ventricular remodeling with echocardiography, in patients with total and subtotal occluded left anterior descending artery (LAD) in the subacute phase of anterior MI.

## Methods

### Study population

Sixty stable patients with subacute anterior wall MI were enrolled in the study. The exclusion criteria included the following: patients who had received thrombolytic theraphy or who had PCI performed in the early term of MI. Also excluded were patients who presented unstable clinic or any findings suggesting ongoing myocardial ischemia, angina at rest, NYHA class III or IV heart failure, shock, a serum creatinine concentration higher than 2.5 mg per deciliter, angiographically significant left main or three-vessel coronary artery disease, any significant stenosis in the right or circumflex coronary artery together with a LAD lesion, history of coronary artery disease, cardiac muscle disease, bundle branch block or atrial fibrillation, hemodynamic and electrical instability. Unsuccessful PCI was also an exclusion criterion in the groups to which PCI was applied.

Conventional coronary angiography was performed with Philips Integris 5000 equipment (Philips Medical Systems, Best, Netherlands) in patients within 1 to 3 days after admission. After obtaining images by standard approaches, each angiogram was interpreted by two independent cardiologists. The coronary lesions were classified as total occlusions or subtotal occlusive lesions. The criterion for total occlusion of the LAD artery was absent antegrade flow, defined as a Thrombolysis in Myocardial Infarction (TIMI) flow grade of 0.

To test baseline myocardial viability, Technetium-99m sestamibi scintigraphy was performed on all patients with an over17-segment model before the patient received the assigned treatment.

Sixty patients were divided into three groups according to the angiographic properties of patients and treatment option. The total-medical group consisted 20 patients who had total occlusion in LAD and received only medical therapy. The total-PCI group consisted 20 patients who had total occlusion in LAD and had PCI performed. Another 20 patients who had subtotal occlusion in LAD and had PCI performed were included in the subtotal-PCI group.

The patients in the total-medical group received optimal medical therapy alone. The patients in the total-PCI and subtotal-PCI groups were assigned to PCI with stent placement and optimal medical therapy. Optimal medical therapy included aspirin, angiotensin converting enzyme inhibitors, beta-blockers, lipid lowering therapy, and clopidogrel before PCI and 4 weeks after PCI in patients who underwent stenting. In the total-PCI and subtotal-PCI groups, PCI was performed at 2-28 days after myocardial infarction. Successful PCI was defined as an open artery with residual stenosis of less than 30% and a TIMI flow grade of 3. The study was approved by the University ethics committee. All the patients were informed about the study, and their written consent forms were obtained.

### Echocardiography

The Echocardiographies were performed by two cardiology specialists with Vivid 7 instruments (GE Medical Systems, Milwaukee, WI, USA), with a 2.5-MHz transducer and harmonic imaging in the cardiology department’s echocardiography laboratory. All echocardiography results were obtained after admission and at the end of the first month after discharge. According to the recommendations of the American Society of Echocardiography [10], all echocardiographic examinations were performed with the patient lying in the left lateral decubitus position, and two-dimensional images were recorded and measured at the apical 4 chambers, 2 chambers, and parasternal long axis views**.** Left ventricular systolic (LVSD) and diastolic diameters (LVDD) were measured by M-mode echocardiography. Left ventricular end diastolic (LVEDV) and end systolic volumes (LVESV) were measured at apical four chambers view. Left ventricular ejection fraction (LVEF) was assessed using the modified biplane Simpson’s method. Left ventricular mass (LV mass) was calculated by the Penn convention [11].

### SPECT Imaging Protocol

To determine the myocardial viability, Technetium-99m sestamibi scintigraphies were performed on all patients with an over 17-segment model after echocardiographic evaluations.

MPS images were analyzed by two experienced nuclear medicine physicians who had no knowledge of all the other data. All enrolled participants underwent a rest protocol using gated SPECT myocardial perfusion imaging with technetium-99m methoxyisobutylisonitrile (Tc-99m MIBI). Before receiving Tc-99m MIBI, patients were given 1 to 2 tablets of sublingual nitroglycerin (0.4 mg) 5 minutes apart and were injected with 740 MBq (20 mCi) of 99mTc-MIBI at rest and gated myocardial perfusion SPECT was performed 45 minutes later.

Gated SPECT MIBI data were acquired in the supine position with the double-head SPECT γ camera (Siemens, e.cam;USA) equipped with a high-resolution low-energy collimator. Thirty-two projection images over 180° noncircular orbit were acquired. Time per projection was 35 s, matrix size 64 x 64 and gating eight frames per cardiac cycle.

Using the e.soft commercial software, transaxial tomograms were generated from gated projection data, reconstructed with filtered back-projected algorithm and reoriented to obtain oblique-angle tomograms paralel to the long and short axes of the left ventricle. The reconstructed data were projected as myocardial tomographic slices in short axis, vertical-long axis and horizontal-long axis views. Gated SPECT MIBI data were then processed and analyzed using 4D-MSPECT software. The myocardium was divided into 17 segments following the American Society of Nuclear Cardiology/ American College of Cardiology/ Amercian Heart Association guidelines [12].

### Statistical analysis

Data are expressed as mean ± SD, or percentage. Comparisons between the groups were carried out using Oneway ANOVA test. To compare the change of measurements between baseline and first month in each group, student paired test was used. The SPSS 15.0 software was used for statistical analysis (Version 15, SPSS Inc., Chicago, IL, USA).

## Results

Mean age was 62.4 ± 11.4 years in the study population (43 men and 17 women). Demographic properties of patients were similar in all groups (Table 1). At the end of the one-month follow-up, two patients died in the total-PCI group and one patient died in the total-medical group.

**Table 1:** Baseline Characteristics of All Patients in Three Groups

	Total-PCI Group (n:18)	Total-Medical Group (n:19)	Subtotal-PCI Group (n:20)	P value
Age	62 ± 12	65 ± 8	59 ± 12	0.2
Female (n,%)	5 (27)	6 (31)	6 (30)	0.9
DM (n,%)	8 (44)	9 (47)	4 (20)	0.1
HT (n,%)	9 (50)	10 (52)	6 (30)	0.3
Smoke (n,%)	11 (61)	10 (52)	12 (60)	0.8
CVD (n,%)	1 (5)	2 (10)	0	0.3
NYHA Class (n,%)				
I	7 (39)	8 (42)	9 (45)	0.9
II	11(61)	11(58)	11(55)	0.9
III- IV	0	0	0	
Blood Pressure (mmHg)				
Diastolic	82 ± 18	85 ± 15	75 11	0.1
Systolic	133 ± 23	137 ± 28	125 ± 22	0.3
Creatinine (mg/dl)	1.0 ± 0.2	1.1 ± 0.4	0.9 ± 0.1	0.1
Lipid profile (mg/dl)				
LDL-C	120 ± 29	116 ± 43	118 ± 34	0.9
HDL-C	39 ± 9	41 ± 10	37 ± 8	0.4
T.CHOL	192 ± 46	183 ± 54	184 ± 42	0.8
TG	165 ± 131	129 ± 89	144 ± 80	0.5

Data expressed as mean ± SD, P < 0.05 was accepted as a statistically significant. DM: diabetes mellitus, HT: hypertension, CVD: cerebrovascular disease, LDL-C: low density lipoprotein, HDL-C: high density lipoprotein, T.CHOL: total cholesterol, TG: triglyceride

The usage rates of medical therapy were similar in three groups. When examining the comparision of necrotic segment number in technetium-99m sestamibi scintigraphy in all three groups; the mean necrotic segment numbers were similar in three groups (Necrotic segment number was 2.22 ± 1.98 in group Total- PCI, 2.78 ± 3.10 in total- medical group and 2.15 ± 2.45 in subtotal- PCI group, p = 0.2). In the total-PCI and subtotal- PCI groups, the average of mean times from AMI to intervention was 5 ± 1 days. In the total-PCI group, no significant difference was found between baseline and first month LVEF and sPAP measurement. There was a borderline significant decrease in E/E’ ratio in the total-PCI group. However, a significant increase was observed in LVDD, LVEDV, LVESV, LAD and LV Mass (Table 2).

**Table 2:** The Change of Echocardiographic Parameters at Baseline and First Month in the Total-PCI Group

	Baseline	First month	p value
LVSD (cm)	3.4 ± 0.4	3.5 ± 0.4	0.1
LVDD (cm)	4.8 ± 0.4	4.9 ± 0.3	0.02
LVESV (mL)	59 ± 27	68 ± 26	0.03
LVEDV (mL)	98 ± 35	114 ± 35	0.01
LVEF (%)	39 ± 8	40 ± 8	0.1
LV Mass (gr)	198 ± 57	218 ± 54	0.005
LAD (cm)	3.6 ± 0.3	3.7 ± 0.2	0.03
sPAP (mmHg)	33 ± 12	34 ± 12	0.5
E/E’	11±5	8±4	0.05

Data expressed as mean ± SD, P < 0.05 was accepted as a statistically significant. LV: Left ventricular LVDD: Left ventricular diastolic diameter, LVSD: Left ventricular systolic diameter, LVEDV: Left ventricular enddiastolic volume, LVESV: Left ventricular endsystolic volume, LVEF: Left ventricular ejection fraction, LVM: Left ventricular mass, LAD: left atrial diameter, sPAP: systolic pulmonary artery pressure, E/E’: ratio of transmitral E velosity to myocardial E velosity

Comparing to the baseline values, LVDD, LVSD, LVESV, LAD, LVEF and sPAP were not significantly changed at the first month in the total-medical group (Table 3). A borderline significant increase was observed in LVEDV and LV Mass at the first month compared to baseline measurement (LVEDV: 113 ± 6 ml vs. 126 ± 6 ml, p: 0.05, LV Mass: 214 ± 15 gr vs. 235 ± 16 gr, p = 0.04, respectively). There was also a significant decrease in E/E’ ratio at first month (Table 3).

**Table 3:** The Change of Echocardiographic Parameters at Baseline and First Month in the Total-Medical Group

	Baseline	First month	p value
LVSD (cm)	3.8 ± 0.5	3.8 ± 0.4	0.5
LVDD (cm)	5.0 ± 0.5	5.0 ± 0.4	0.3
LVESV (mL/mm2)	75 ± 23	82 ± 25	0.1
LVEDV (mL/mm2)	113 ± 27	126 ± 29	0.05
LVEF (%)	35 ± 9	36 ± 9	0.2
LV Mass (gr)	214 ± 67	235 ± 73	0.04
LAD (cm)	3.7 ± 0.4	3.7 ± 0.4	0.9
sPAP (mmHg)	38 ± 11	36 ± 9	0.2
E/E’	10±4	8±3	0.02

Data expressed as mean ± SD, P < 0.05 was accepted as a statistically significant. LV:Left ventricular LVDD: Left ventricular diastolic diameter, LVSD: Left ventricular systolic diameter, LVEDV: Left ventricular enddiastolic volume, LVESV: Left ventricular endsystolic volume, LVEF: Left ventricular ejection fraction, LVM: Left ventricular mass, LAD: left atrial diameter, sPAP: systolic pulmonary artery pressure, E/E’: ratio of transmitral E velosity to myocardial E velosity

No significant difference was found at first month comparing to the baseline values in all parameters except sPAP in the subtotal-PCI group. There was a significant decrease in sPAP at the first month (Table 4).

**Table 4:** The Change of Echocardiographic Parameters at Baseline and First Month in the Subtotal-PCI Group

	Baseline	First month	p value
LVSD (cm)	3.7 ± 0.5	3.8 ± 0.5	0.5
LVDD (cm)	5.0 ± 0.5	5.1 ± 0.5	0.2
LVESV (mL/mm2)	66 ± 30	68 ± 32	0.4
LVEDV (mL/mm2)	113 ± 40	115 ± 38	0.5
LVEF (%)	42 ± 9	42 ± 9	0.3
LV Mass (gr)	220 ± 59	231 ± 69	0.07
LAD (cm)	3.7 ± 0.5	3.7 ± 0.4	0.5
sPAP (mmHg)	32 ± 10	29 ± 10	0.02
E/E’	9 ± 4	8 ± 3	0.1

Data expressed as mean ± SD, P < 0.05 was accepted as a statistically significant. LV:Left ventricular LVDD: Left ventricular diastolic diameter, LVSD: Left ventricular systolic diameter, LVEDV: Left ventricular enddiastolic volume, LVESV: Left ventricular endsystolic volume, LVEF: Left ventricular ejection fraction, LVM: Left ventricular mass, LAD: left atrial diameter, sPAP: systolic pulmonary artery pressure, E/E’: ratio of transmitral E velosity to myocardial E velosity

**Figure 1:**
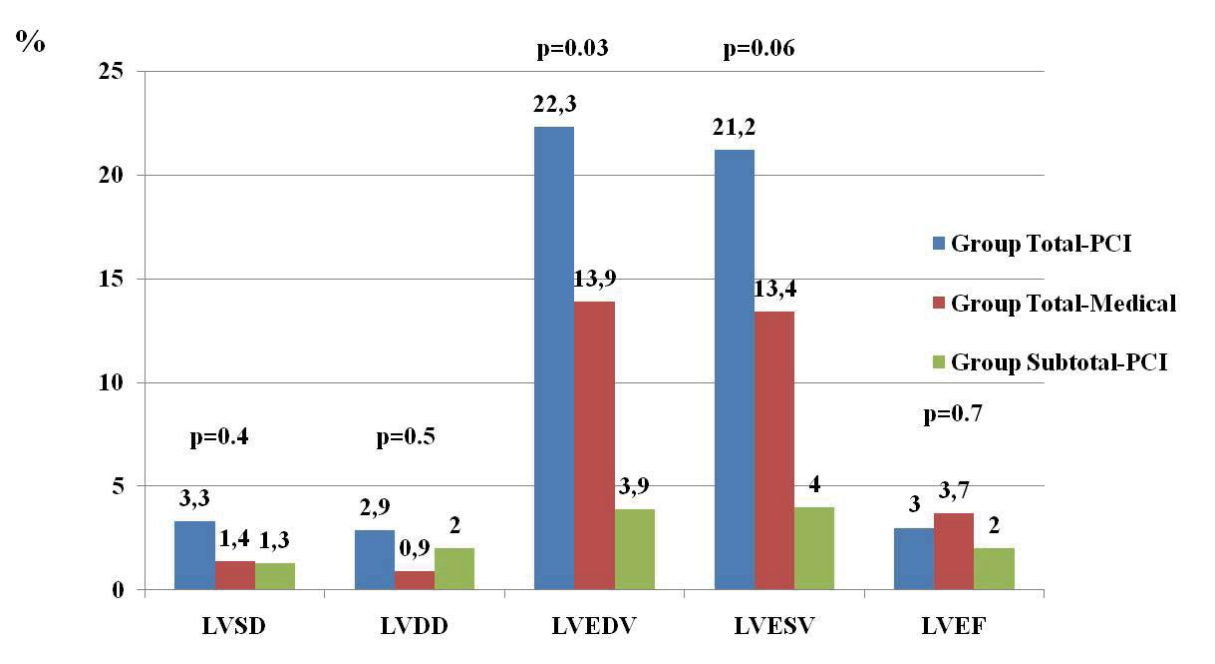
Percentage changes of echocardiographic parameters in three groups. P < 0.05 was accepted as a statistically significant. LV:Left ventricular LVDD: Left ventricular diastolic diameter, LVSD: Left ventricular systolic diameter, LVEDV: Left ventricular enddiastolic volume, LVESV: Left ventricular endsystolic volume, LVEF: Left ventricular ejection fraction.

While the percentages of increase in LVEDV and LVESV were significant in the total-medical group (22.3% and 21.2 %, respectively, p = 0.03), there were borderline significant in the total-medical group (13.9% and 13.4%, respectively, p = 0.06). The lowest and non- significant changes in left ventricular volumes were seen in the subtotal-medical group. The increases in LV diameters and EF at the first month comparing to baseline were not significant in all three groups (Fig. 1).

## Discussion

Using PCI to treat infarct-related artery occlusion in stable patients is still a controversial issue. We assessed the effect of PCI and optimal medical therapy in patients with total or subtotal occlusion in an infarct related LAD artery. Present results showed that PCI applied to total occlusion on infarct-related LAD caused higher LV remodeling when compared to medical therapy for total occlusion and PCI for total occlusion. These results emphasize the importance of avoiding invasive intervention in stable patients who had total occlusion in the infarct-related LAD artery.

For many years, open artery hypothesis was defended as treatment that would improve LV functions and reduce LV remodeling after late reperfusion [13, 14]. However, in recent years, large randomized studies demonstrated no clinical benefit from PCI for a totally occluded infarct-related artery in asymptomatic patients who had hemodynamically and electrically stable clinical findings and who did not have evidence of severe ischemia [4, 8, 9].** **Based on these results, current guidelines do not recommend PCI for a totally occluded infarct-related artery in asymptomatic patients [15]. However, the effect of PCI in patients with subacute total occlusion on left ventricle function is controversial. From the OAT study, TOSCA II shows that PCI with stenting of a persistently occluded IRA in the subacute phase after myocardial infarction has no effect on the LV ejection fraction [4]. After the TOSCA study, Abbate et al. published a meta-analysis of randomized trials comparing PCI of the infarct-related artery (IRA) and medical therapy in patients >12 h after acute myocardial infarction [16]. They found that PCI of the IRA performed late (12 h to 60 days) after AMI was associated with significant improvements in cardiac function. In detailed analysis, patients’ characteristics in both the TOSCA study and other studies in the meta-analysis were considerably heterogeneous. In the TOSCA study, the infarct-related artery in only 30% patients was the LAD artery. Also IRA TIMI flow grade 0 in 79% and single vessel disease was 81.5%. Also, the timing of PCI, duration of follow up, initial diagnosis, total or subtotal occlusion rate changed in each study in the meta-analysis. Therefore, to make a clinical decision based on these studies is quite difficult and obscure. In our study, the infarct-related artery was only the proximal LAD and one patient who had another lesion from a culprit lesion was excluded. IRA TIMI flow grade was 0 in all patients. We aimed to include highly specific patient population to the study. So, we aimed to access the accuracy of opinions about PCI or medical therapy in stable patients with LAD total occlusion after MI.

The primary important result of our study is that PCI performed on the infarct-related LAD total occlusion in stable patients in late phase after anterior myocardial infarction is associated with an increase in LV remodeling at the end of the first month. In the PCI-to0cclusion group, LVDD, LVEDV, LVESV and LVMI were significantly increased. However, no significant change was observed in these parameters in the only medically treated patients. When analyzed according to the rate of percent variation, the most prominent unfavorable result was shown in the total-PCI group. The percent variations on LVEDV and LVESV were 7.8% and 6.1% in patients who received medical therapy. However, in the total-PCI group, these values were 12.9% and 11.1%. These results showed that some degree of LV remodeling occurred in patients with infarct-related total occlusion after MI. However, these negative findings raise questions. A conflict results about the management of patients with total occlusion in the post-MI period. Our study population was small but included a very specific group. Patients had only proximal LAD total occlusion and TIMI grade flow 0. Therefore, our results clearly show that applying PCI to total occlusion after MI causes more prominent LV remodeling compared to only medical therapy.

On the other hand, we analyzed the effect of PCI on LV function in the subtotal LAD occlusion group in the post-MI period. To the best of our knowledge, no clinical study compares the effect of PCI on patients with total and subtotal infarct-related MI. Thus, we aimed to compare the echocardiographic results of PCI in total occlusion and subtotal occlusion MI patients. At the end of the first month, a significant difference in LVEF was not observed in patients who received PCI to subtotal occlusion compared to the total-PCI group. However, increases in LV volume and diameter were minimal. While increases in LVEDV and LVESV were 12.9% and 11.1%, these rates were 1.1% and -0.09% in the subtotal-PCI group. According to these results, we suggest that PCI for subtotal occlusion of infarct-related LAD prevents left ventricular remodeling.

### Limitations

Our study’s most important limitation was the small number of patients. Follow-up time was only one month. Therefore, large and long-term follow-up studies are needed. Also we included only LAD total occlusion.

### Conclusion

This study shows that in stable patients, PCI for total occlusion had no echocardiographic benefit in the subacute phase of anterior MI. Moreover PCI caused an increase in LV remodeling. PCI for subtotal occlusion in the subacute phase of anterior MI may prevent left ventricular remodeling.
